# Integration of metabolomics and transcriptomics provides insights into enhanced osteogenesis in *Ano5^Cys360Tyr^* knock-in mouse model

**DOI:** 10.3389/fendo.2023.1117111

**Published:** 2023-01-20

**Authors:** Hongyu Li, Sirui Liu, Congcong Miao, Yan Lv, Ying Hu

**Affiliations:** Beijing Institute of Dental Research, Beijing Stomatological Hospital, Capital Medical University, Beijing, China

**Keywords:** Gnathodiaphyseal dysplasia, ANO5, metabolomics, transcriptomics, cell proliferation, calcium, osteogenesis

## Abstract

**Introduction:**

Gnathodiaphyseal dysplasia (GDD; OMIM#166260) is a rare autosomal dominant disorder characterized by diaphyseal sclerosis of tubular bones and cemento-osseous lesions in mandibles. GDD is caused by point mutations in the *ANO5* gene. However, the mechanisms underlying GDD have not been disclosed. We previously generated the first knock-in mouse model for GDD expressing a human mutation (p.Cys360Tyr) in *ANO5* and homozygous *Ano5* knock-in (*Ano5^KI/KI^*) mice exhibited representative traits of human GDD especially including enhanced osteogenesis.

**Methods:**

Metabolomics and transcriptomics analyses were conducted for wildtype (*Ano5^+/+^*) and *Ano5^KI/KI^* mature mouse calvarial osteoblasts (mCOBs) grown in osteogenic cultures for 14 days to identify differential intracellular metabolites and genes involved in GDD. Subsequently, related differential genes were validated by qRT-PCR. Cell proliferation was confirmed by CCK8 assay and calcium content in mineral nodules was detected using SEM-EDS.

**Results:**

Metabolomics identified 42 differential metabolites that are primarily involved in amino acid and pyrimidine metabolism, and endocrine and other factor-regulated calcium reabsorption. Concomitantly, transcriptomic analysis revealed 407 differentially expressed genes in *Ano5^KI/KI^* osteoblasts compared with wildtype. Gene ontology and pathway analysis indicated that *Ano5^Cys360Tyr^* mutation considerably promoted cell cycle progression and perturbed calcium signaling pathway, which were confirmed by validated experiments. qRT-PCR and CCK-8 assays manifested that proliferation of *Ano5^KI/KI^* mCOBs was enhanced and the expression of cell cycle regulating genes (*Mki67*, *Ccnb1*, and *Ccna2*) was increased. In addition, SEM-EDS demonstrated that *Ano5^KI/KI^* mCOBs developed higher calcium contents in mineral nodules than *Ano5^+/+^* mCOBs, while some calcium-related genes (*Cacna1*, *Slc8a1*, and *Cyp27b1*) were significantly up-regulated. Furthermore, osteocalcin which has been proved to be an osteoblast-derived metabolic hormone was upregulated in *Ano5^KI/KI^* osteoblast cultures.

**Discussion:**

Our data demonstrated that the *Ano5^Cys360Tyr^* mutation could affect the metabolism of osteoblasts, leading to unwonted calcium homeostasis and cellular proliferation that can contribute to the underlying pathogenesis of GDD disorders.

## 1 Introduction

Gnathodiaphyseal dysplasia (GDD; OMIM#166260) is a rare skeletal disorder mainly characterized by cemento-osseous lesions of mandibles and tubular bone fragility (1). GDD is inherited in an autosomal dominant pattern or sporadically occurs. Tsutsumi used linkage analysis to identify that this syndrome is associated with mutations of *GDD1*, also referred to as *Anoctamin 5* (*ANO5*; *TMEM16E*) that is mapped to chromosome 11p14.3-15.1 (2). The protein encoded by *ANO5* belongs to the TMEM16/anoctamin protein family and is widely expressed in skeletal muscle and bone tissues (3, 4). Unlike other TMEM16 family members that are localized within plasma membranes, ANO5 is predominantly located in intracellular vesicles, including in the endoplasmic reticulum, Golgi apparatus, and endosomes (3). Thus, it remains controversial whether ANO5 exhibits activities related to calcium-activated chloride channels (CaCCs) or phospholipid scrambling (5–8).

Heretofore, GDD-related *ANO5* missense mutations have been identified at seven positions, including p.Arg215Gly, p.Cys356Gly, p.Cys356Arg, p.Cys356Tyr, p.Cys356Phe, p.Cys360Tyr, p.Ser500Phe, p.Thr513Ile, p.Gly518Glu, and Arg597Ile (3, 9–17). Enhanced bone formation is a momentous clinical feature of patients with GDD, as evinced by radiographic examinations revealing relatively elevated bone mineral density (BMD) and laboratory data documenting high serum levels of alkaline phosphatase (ALP). Consequently, greater attention has been paid to the effects of *ANO5* mutations on osteoblast functions in recent years. *Ano5* silencing in MC3T3 promoted the formation of bone mineral nodules and the expression of osteoblast differentiation markers, including the runt-related transcription factor 2 (Runx2), collagen I (Col1a1), and osteocalcin (Ocn) (13). In addition, *Ano5* deletion in mice replicated some typical traits of human GDD, including elevated serum ALP activity, high BMD and bone mineral content (BMC) of mandibles and diaphyseal of long bones, and increased osteogenesis (18). However, the specific mechanisms underlying the aberrant bone formation in GDD remain unclear. Thus, there is an urgent need to provide a systematically reliable observation of osteogenic alternation caused by GDD-related mutations.

Bone is a multitasking tissue with mechanical, hematopoietic, and metabolic functions, which depends on the precise cooperation of osteoblasts and osteoclasts. Various metabolic pathways are indispensable for maintaining bone tissue (19). Osteoblasts require substantial amounts of energy during new bone formation and remodeling. Clinical disorders related to substrate availability, like diabetes mellitus, anorexia nervosa, and aging, dysregulate osteogenesis ultimately leading to osteoporosis (20). Many lines of evidence have shown that osteoblasts secrete endocrine factors including OCN, sclerostin (SOST), and fibroblast growth factor 23 (FGF23) that connect the metabolic requirements of bone formation with global energy balance (20, 21). Beyond aerobic glycolysis, which produces 80% adenosine triphosphate (ATP) through utilizing glucose, glutamine *via* the TCA cycle and fatty acids *via* oxidative phosphorylation are also important fuel sources for osteoblasts and necessary for generating metabolic intermediates to support matrix protein synthesis (20). Furthermore, it has been proposed that higher uptake and utilization of protein, a potential energy source in addition to the above, is beneficial for bone health, as mediated by its role in forming bone matrix structures and stimulating osteoblast activity (22, 23). Ionic calcium metabolism is one of the mineral components in the extracellular matrix (ECM) and is required for bone matrix formation, the synthesis of mineral scaffolding ECM, and mineral crystal formation (24). Metabolomics has become popular in recent years for understanding bone diseases including osteonecrosis, osteoarthritis, intervertebral disc degeneration, and osteoporosis due to the synchronized relationships between metabolism and bone development (25, 26). Indeed, metabolomics is an emerging tool for biomarker identification *via* comprehensive and systematic profiling of low molecular weight metabolites that are the substrates and products of metabolism driving essential cellular functions involved in signal transduction, cellular proliferation, ion transport, and energy production (27). Concomitantly, RNA-Seq methods that allow profiling of whole transcriptome and therefore revealing alterations in entire signaling networks, have also been routinely used in skeletal biology research (28).

We previously reported a Chinese GDD family carrying the p.Cys360Tyr mutation in *ANO5*. Further, an *Ano5^Cys360Tyr^* knock-in mouse model was successfully established that resembled some phenotypes of GDD patients and exhibited enhanced osteogenesis (29). In the present study, RNA-seq and metabolomics analyses were used to explore differentially expressed genes and intracellular metabolites of mature mouse calvarial osteoblasts (mCOBs) after 14-day osteogenic cultures from *Ano5^Cys360Tyr^* compared with *Ano5^+/+^* mice, and the underlying metabolic pathways were predicted followed by verification with functional experiments.

## 2 Materials and methods

### 2.1 Generation of the *Ano5^KI/KI^* mouse model

The *Ano5^KI/KI^* mouse model carrying a Han GDD mutation was generated using CRISPR Cas9 genomic editing technology and by introducing a transformation of cysteine into tyrosine at codon 360 of *Ano5*. C57BL/6 female mice and KM mouse strains were used as embryo donors and pseudo-pregnant foster mothers, respectively, and were purchased from the Beijing Vital River Laboratory Animal, Co., Ltd. Genotyping of the *Ano5* knock-in mice was verified by PCR amplification and Sanger sequencing (forward primer: 5’-GCTTAGGTCTTCTACATCGGGCTGT-3’ and reverse primer: 5’-ATCCCCATGAAGAGCGCAAAGAACA-3’). Details related to generation and genotype identification of the knock-in mouse model were previously published (29). Mice were housed in a pathogen-free environment under 12 hours light-dark cycles and given standard food *ad libitum*. All animal experimentation protocols were approved by the Institutional Animal Care and Use Committee of the Beijing Stomatological Hospital (the approval number: KQYY-201611-001).

### 2.2 mCOB isolation and cultures

mCOBs from *Ano5^+/+^* and *Ano5^KI/KI^* mice were isolated from postnatal 24 hour old littermates and cultured in DMEM after adding 20% fetal bovine serum (FBS; Gibco, USA) until reaching 80% confluence. mCOBs at the third passage were used for osteoblast differentiation in osteogenic medium, as previously described. Mature osteoblasts represent mCOBs after 14 days of osteoblast differentiation. Cultivation medium was exchanged every two days.

### 2.3 Sample preparation and Ultra Performance Liquid Chromatography-Tandem Mass Spectrometry (UPLC-MS/MS) detection

2×10^7^ mCOBs/well from *Ano5^+/+^* (n=6) and *Ano5^KI/KI^* mice (n=8) after 14 days of osteogenic induction were collected into centrifuge tube after washing three times with PBS buffer and all subsequent operations were carried out on ice. Cell extracts (500 μl comprising 80% methanol and internal standards, 20% H_2_O) were added and vortexed for 3 minutes to achieve complete sample suspension. Samples were placed on liquid nitrogen for 5 minutes to achieve rapid freezing, then thawed on dry ice and ice for 5 min each, and then mixed by vortex for 2 minutes. The entire procedure was repeated in triplicate. Centrifugation was subsequently conducted for 10 min at 12,000 rpm/min and 4°C. Supernatants from samples (300 μl) were transferred into another centrifuge tube and incubated at -20°C for 30 min. Insoluble fragments were discarded by centrifugation at 12,000 rpm/min for 3 min and 4°C for 3 min and then 200 μl of supernatant was removed into the liner column of the sampling bottle for LC-MS analysis.

Chromatographic separation was firstly performed in a ThermoUltimate 3,000 system equipped with a Waters ACQUITY UPLC HSS T3 C18 column (100 × 2.1 mm, 1.8 μm, Waters) maintained at 40°C. Gradient elution analysis was carried out with 0.1% formic acid in water (A) and 0.1% formic acid in acetonitrile (B) at a flow rate of 0.4 ml/min. The injection volume of each sample was 2 μl. A gradient of water/solvent B (v/v) was used as follows: 0 min, 95%/5%; 11.0 min, 10%/90%; 12.0 min, 10%/90%; 12.1 min, 95%/5%; 14 min: 95%/5%. Liquid chromatography was then accomplished with a Waters ACQUITY UPLC BEH Amide column (100 × 2.1 mm, 1.7 μm, Waters) maintained at 40°C. Gradient elution was proceeded in water with 20 mM ammonium formate and 0.4% ammonia solution (A) and acetonitrile (B) at a flow rate of 0.4 ml/min. The injection volume of each sample also was 2 μl. A gradient of water/acetonitrile (v/v) was used as follows: 0 min, 10%/90%; 9.0 min, 40%/60%; 10.0 min, 60%/40%; 11.0 min, 60%/40%; 11.1 min, 10%/90%; 15.0 min, 10%/90%. The mass spectroscopy acquisition conditions included an electrospray ionization (ESI) temperature of 500°C, positive voltage of 5,500 V, a negative voltage of -4,500 V, the ion source gas I (GS I) at 55 psi, the GS II at 60 psi, the curtain gas (CUR) at 25 psi; and with the high collision-activated ionization (CAD) parameter. Full scan detection was performed in triple quadrupole (Qtrap) mode according to the optimized declustering potential (DP) and collision energy (CE).

### 2.4 Metabolomics analysis

Raw UPLC-MS/MS data were processed using Analyst (version 1.6.3). Integration and correction of chromatographic peaks were conducted using the MultiaQuant software package. Principal component analysis (PCA) was carried out using the base R software package (version 3.5.1). Intracellular metabolites significantly differential between groups were determined based on variable importance in projection (VIP) values ≥1 and *p*-values < 0.05. VIP values extracted from the orthogonal projection to latent structures discriminant analysis (OPLS-DA) results and associated score plots and permutation plots, as generated with the R package MetaboAnalystR (version 1.0.1). In order to avoid overfitting, a permutation test with 200 permutations was performed. Hierarchical cluster analysis (HCA) was conducted using the R package heatmaply (version 1.2.1) and ComplexHeatmap (version 2.7.1.1009). Pearson correlation coefficients between sample profiles were calculated using the cor function of R (version 3.5.1) and were visualized as heatmaps. Identified metabolites were annotated using the KEGG compound database and then mapped to the KEGG pathway database. Significantly enriched pathways were identified using the *p*-value from hypergeometric tests for a given set of metabolites.

### 2.5 RNA isolation and RNA-Seq analysis

Total RNAs of 5×10^6^ mCOBs from *Ano5^+/+^* and *Ano5^KI/KI^* mice (n=3 per group) cultured after 14 days of osteogenic induction were extracted using the Trizol reagent (Ambion, Thermo Fisher Scientific, USA). The integrity of the total RNA was evaluated with an Agilent 2100 Bioanalyzer and with agarose gel electrophoresis (28S:18S values ≥1, RNA integrite number (RIN) values ≥7), along with purity and quantification analysis with a Nanodrop spectrophotometer (concentration ≥ 50 ng/μl, 260/280 absorbance ≥1.8, 28S/18S ≥1). mRNA was enriched using magnetic beads with Oligo (dT) and then fragmented. One-strand cDNA was synthesized from the mRNA templates using reverse transcription with random hexamers. Double-stranded cDNA was then synthesized by adding dNTPs and DNA polymerase I. AMPure XP beads were used to purify and select desired fragment size ranges of double-stranded cDNA, and finally PCR amplification was performed to construct cDNA libraries. Subsequently, qPCR was used to measure RNA concentrations (> 4 nM). Paired-end sequencing with a read length of 200-300 bp was then conducted on the Illumina HiSeqTM2500/4000 platform (Illumina Inc., San Diego, CA, United States).The experiments were carried out in triplicates and mCOBs cultures from at least three different mice.

### 2.6 Transcriptomics analysis

Transcript expression levels are expressed as fragments per kilobase of exon model per million mapped reads (FPKM). Genes or transcripts with mean FPKM values > 1.0 are considered to be expressed in the group for statistical analysis. The HTSeq software package was used to analyze the expression levels in each sample using the UNION model. The Trimmomatic software program (version 0.33) was used to perform quality control of reads based on RNA-seq. Sequence reads were mapped to the reference genome using the STAR program (version 2.5.2b) software package. The DEGSeq 1.12.0 and DESeq package for R (version 1.10.1) were used to identify differentially expressed genes (DEGs) in *Ano5^KI/KI^* mCOBs compared to *Ano5^+/+^* mCOBs. The negative binomial distribution and Benjamini–Hochberg methods were used to calculate *p* and FDR values, respectively. A total of 407 DEGs were filtered out under a reasonable threshold and effective criteria of fold change >1.2 and *p*_adj_ < 0.05. The GOseq (version 1.22) and KOBAS (version 2.0) packages for R were used to conduct Gene ontology (GO) and KEGG pathway enrichment to identify the physiological associations of DEGs. Significantly enriched items and pathways are identified using q values (corrected *p*-values) that were calculated by hypergeometric tests with BH correction.

### 2.7 Scanning Electron Microscopy with X-ray Energy-Dispersive Spectroscopy (SEM-EDS)

mCOBs from *Ano5^+/+^* and *Ano5^KI/KI^* mice were cultured on 24-well glass coverslips in osteogenic induction medium for 21 days. Samples (n=5 per group) were fixed with 2.5% glutaraldehyde and dehydrated with an ethanol gradient. The micromorphology of the nodules was then examined by SEM (TESCAN S9000X), while calcium and phosphate contents were analyzed by EDS using a MERLIN VP Compact system (Zeiss, Germany) in the UH-resolution scan mode under an acceleration voltage of 5 kV.

### 2.8 Cell proliferation assay

The proliferative ability of mCOBs was tested using a cell counting kit-8 (CCK-8) assay (Dojindo, Tokyo, Japan) according to the manufacturer’s instructions. Cells were plated at a density of 5 × 10^3^ cells/well in 96-well plates. Complete exchange of the medium was conducted at days 1, 3, and 4 using serum-free medium containing the CCK-8 reagent, followed by incubation with the cells at 37°C for 2 hours and OD measurements at 450 nm using a microplate reader. Meanwhile, the viability of mCOBs after 14 and 21 days of osteoblast differentiation was also measured. Cell proliferation rate was calculated as follows: cell proliferation rate = ((OD_treat-_OD_Blank_) - (OD_12h-_OD_Blank_))/(OD_12h-_OD_Blank_) x 100%.

### 2.9 Cell cycle analysis

*Ano5^+/+^* and *Ano5^KI/KI^* mCOBs were seeded at a density of 3×10^5^ cells/well into 6-well plates and then cultured with α-MEM for 24 hours or with 14 day osteoblast differentiation. Cells were then digested and resuspended in a centrifuge tube and the supernatant was discarded after centrifugation at 1,000 *g*/minfor 5 min. After washing twice with cooled PBS, cells were fixed in 70% methyl alcohol at 4°C overnight. Cells were then incubated with 500 μL propidium iodide (PI) staining buffer for 30 min in the dark at 37°C. A BD Accuri C6 flow cytometer was applied to detect red fluorescence at an excitation wavelength of 488 nm, while ModFitLT V3.2 was utilized to analyze cell cycle distributions (G1, S, and G2).

### 2.10 Biochemistry analysis

Serum was collected from the retroorbital veins of 16 week old *Ano5^+/+^* and *Ano5^KI/KI^* male mice (n=10) using a glass capillary. After allowing to naturally solidify at room temperature, supernatants were collected after centrifugation at 1,000 *g* for 20 min. In addition, cell-free extracts and corresponding culture supernatants from *Ano5^+/+^* and *Ano5^KI/KI^* mCOBs were acquired at days 0 and 14. Calcitriol levels were determined using a calcitriol ELISA Kit (Sango, Shanghai, China) and FGF23 levels were examined with Mouse FGF23 ELISA Kit (Beyotime, Shanghai, China) according to the manufacturer’s instructions. OD values were immediately measured at 450 nm with a microplate reader.

### 2.11 Western blot analysis

mCOBs after 14 days of osteogenic induction were collected and lysed in RIPA buffer, as previously described (29). The protein concentrations in each sample were measured using Bradford assays with Coomassie brilliant blue G-250 (Bio-Rad, California, USA). A total of 20 μg protein was subjected to sodium dodecyl sulfate polyacrylamide gel electrophoresis (SDS‐PAGE). The SDS membranes were blocked with 5% nonfat milk for 1 hour and incubated with primary OCN antibodies overnight (DF12303, Affinity Biosciences, Beijing, China). ACTB (Abclonal, Wuhan, China) was examined as the housekeeping reference protein. After incubation for 1 hour with horseradish peroxidase (HRP)-conjugated anti-rabbit secondary antibody at a dilution of 1:5,000, band signals were detected using a Bio-Rad imaging system (Bio-Rad, USA) with NcmECL High (NCM Biotech, Jiangsu, China). The Image lab software program (Bio-Rad, USA) was utilized to perform relatively quantitative analysis of protein levels.

### 2.12 Analysis of mRNA expression levels using RT-qPCR

Total RNA was extracted using the TRIzol reagent (Ambion, Life Technologies, USA), and RNA quality was subsequently assessed using the Infinite M200 PRO NanoQuant absorbance microplate reader (TECAN, Chapel Hill, NC, USA). Reverse transcription was conducted with a SuperRT cDNA Synthesis Kit (CWbio, Beijing, China) and real-time quantitative reverse transcriptase-polymerase chain reactions (qRT-PCR) were performed using the Ultra SYBR Mixture with low ROX (CWbio), as previously described (29). Gene expression was calculated using the 2^−ΔΔCt^ method and *Actb* expression was used as the internal control. PCR primer sequences used in qPCRs are listed in **Supplementary Table 1** and all assays were performed in triplicate.

## 3 Results

### 3.1 Metabolic profiling of *Ano5^Cys360Tyr^* mature mCOBs

**Figure 1:**
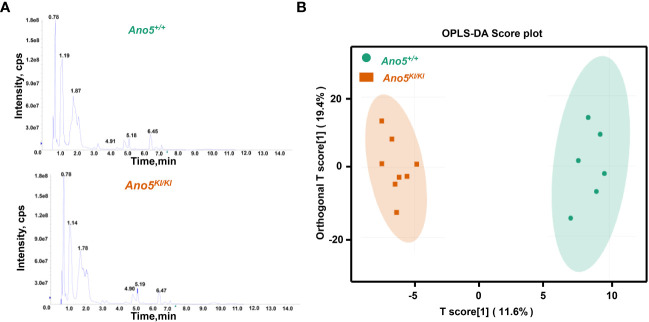
Multivariate statistical analysis of metabolite profiles. **(A)** Total ion current (TIC) diagram of *Ano5^+/+^* (upper panel) and *Ano5^KI/KI^* (lower panel) mCOBs. The X-axis indicating retention time (Rt, min) for metabolite detection and the Y-axis representing ion flow intensity (CPS, count per second); **(B)** OPLS-DA multivariate statistical analysis. Separation of the *Ano5^+/+^* and *Ano5^KI/KI^* groups occurs in the horizontal (T1) direction, while the vertical (orthogonal T1) axis reflects intra-group variability.

A widely targeted metabolomics analysis was conducted using UPLC-MS/MS to explore critical biochemical compounds involved in GDD due to the *Ano5^Cys360Tyr^* mutation. The stability and precision of the data were verified based on the typical base peak intensity chromatograms of the *Ano5^+/+^* and *Ano5^KI/KI^* mCOBs cultured in osteogenic medium for 14 days (**Figure 1A**). The influence of the *Ano5^Cys360Tyr^* mutation on the metabolic patterns of mature mCOBs was analyzed using OPLS-DA model and the result revealed that *Ano5^KI/KI^* mCOBs metabolite profiles were clearly distinguished from the wildtype profiles (**Figure 1B**).

**Figure 2:**
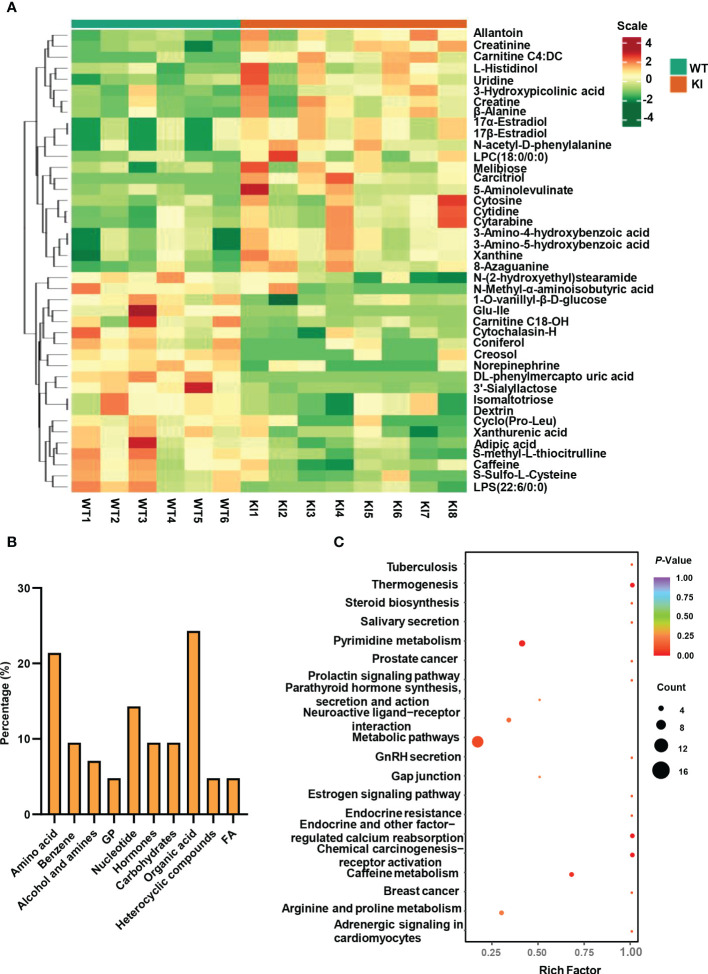
Metabolite analysis of *Ano5^+/+^* and *Ano5^KI/KI^* mCOBs. **(A)** Heat map showing abundances of metabolites with significant differences between *Ano5^KI/KI^* and *Ano5^+/+^* mCOBs. Each row represents a sample, and each column represents a metabolite. Orange shows high expression levels and green represents low expression levels. (WT: *Ano5^+/+^*; KI: *Ano5^KI/KI^*); **(B)** Bar plot of classification of detected metabolites into major functional classes; **(C)** Metabolic pathways enriched in *Ano5^KI/KI^* mCOBs compared to Ano5^+/+^ mCOBs. Each bubble represents a metabolic pathway and the size indicates the number of associated metabolites. Different levels of significance are symbolled by the color gradient.

Significantly altered metabolites were identified *via* VIP values from the OPLS-DA analysis using the strict threshold of VIP > 1 and *p*-values < 0.05 from independent Student’s *t*-tests to identify significantly different metabolites. A total of 20 down-regulated and 22 up-regulated metabolites were screened out in *Ano5^KI/KI^* mCOBs compared with wildtype cultures (**Figure 2A** and **Table 1**). The 20 most differential metabolites based on |Log_2_FoldChange| (|Log_2_Fc|) values were further scrutinized and comprised calcitriol, carnitine C4:DC, 17a-estradiol, 17β-estradiol, N-acetyl-D-phenylalanine, cytosine, cytidine, DL-phenylmercapto uric acid, and cyclo (Pro-Leu) (**Figure S1**). To visualize strengths of correlations across differential metabolites, Pearson correlation analysis was conducted and indicated that 31 metabolites were closely associated with calcitriol (|r| > 0.5) and 30 with carnitine C4:DC (|r| > 0.5), suggesting that these two metabolites considerably affected metabolic disturbances in *Ano5^Cys360Tyr^* osteoblasts (**Figure S2**).

**Table 1:** Significantly differential metabolites in *Ano5^Cys360Tyr^ vs* wild type mCOBs.

Metabolites names	Formula	Index	VIP	*p* value	Log_2_Fc	Type
DL-phenylmercapto uric acid	C11H13NO3S	MADN0501	2.922	0.000	-9.897	down
Glu-Ile	C11H20N2O5	MADP0388	2.482	0.024	-3.725	down
Creosol	C8H10O2	MEDP0745	2.167	0.002	-2.036	down
3’-Sialyllactose	C23H39NO19	MEDN0539	2.638	0.006	-1.985	down
Norepinephrine	C8H11NO3	MEDN0180	1.920	0.002	-1.750	down
Coniferol	C10H12O3	MEDP1237	1.881	0.011	-1.745	down
N-Methyl-α-aminoisobutyric acid	C5H11NO2	MEDP1177	1.963	0.020	-1.653	down
Cyclo(Pro-Leu)	C11H18N2O2	MEDP1919	1.795	0.042	-1.189	down
Carnitine C18-OH	C25H49NO5	MEDP1531	1.863	0.047	-1.000	down
S-methyl-L-thiocitrulline	C7H15N3O2S	MEDP2267	2.063	0.008	-0.884	down
Isomaltotriose	C18H32O16	MEDN1758	1.605	0.034	-0.712	down
dextrin	C18H32O16	MEDN1757	1.605	0.034	-0.712	down
Cytochalasin-H	C30H39NO5	MEDN1613	1.848	0.012	-0.700	down
Adipic Acid	C6H10O4	MADN0083	2.075	0.024	-0.576	down
1-O-vanillyl-β-D-glucose	C14H18O9	MEDN1231	1.803	0.006	-0.562	down
Xanthurenic Acid	C10H7NO4	MADP0258	1.726	0.014	-0.560	down
Caffeine	C8H10N4O2	MEDP1900	1.740	0.023	-0.473	down
S-Sulfo-L-Cysteine	C3H7NO5S2	MADP0042	1.731	0.040	-0.442	down
LPS (22:6/0:0)	C28H44NO9P	MEDN0356	2.362	0.006	-0.408	down
N-(2-hydroxyethyl) stearamide	C20H41NO2	MEDP1040	1.608	0.028	-0.371	down
LPC (18:0/0:0)	C26H54NO7P	MEDP1337	1.679	0.033	0.192	up
Xanthine	C5H4N4O2	MADN0028	2.060	0.011	0.369	up
8-Azaguanine	C4H4N6O	MADP0270	2.046	0.012	0.383	up
3-Amino-4-Hydroxybenzoic Acid	C7H7NO3	MADN0411	1.942	0.018	0.385	up
3-Amino-5-hydroxybenzoic acid	C7H7NO3	MADN0524	1.942	0.018	0.385	up
Uridine	C9H12N2O6	MADP0076	2.119	0.004	0.435	up
Allantoin	C4H6N4O3	MADN0010	2.216	0.003	0.465	up
Creatinine	C4H7N3O	MADP0093	2.417	0.000	0.485	up
Creatine	C4H9N3O2	MADN0086	1.643	0.047	0.564	up
β-Alanine	C3H7NO2	MADN0011	1.924	0.011	0.652	up
L-Histidinol	C6H11N3O	MEDN1890	1.570	0.036	0.708	up
Melibiose	C12H22O11	MEDP1990	1.535	0.044	0.727	up
3-Hydroxypicolinic acid	C6H5NO3	MADN0318	1.704	0.048	0.729	up
5-Aminolevulinate	C5H9NO3	MEDP2002	1.891	0.040	0.848	up
Cytarabine	C9H13N3O5	MEDP1295	1.804	0.030	0.918	up
Cytidine	C9H13N3O5	MADP0065	1.889	0.020	1.017	up
Cytosine	C4H5N3O	MADP0067	1.491	0.041	1.074	up
N-acetyl-D-phenylalanine	C11H13NO3	MADP0531	1.951	0.013	1.414	up
17a-Estradiol	C18H24O2	MEDP1620	1.978	0.011	1.448	up
17β-Estradiol	C18H24O2	MEDP1621	1.978	0.011	1.448	up
Carnitine C4:DC	C11H19NO6	MEDP1434	2.634	0.000	3.399	up
Calcitriol	C27H44O3	MEDP1194	2.920	0.001	11.640	up

The 42 differential metabolites belonged to ten classes including amino acid (21.4%), nucleotide (14.3%), organic acid (14.3%), benzene and substituted derivatives (9.5%), carbohydrates (9.5%), hormones and hormone related compounds (9.5%), alcohol and amines (7.1%), fatty acid (FA, 4.8%), glycerophospholipid (GP, 4.8%) and heterocyclic compound (**Figure 2B**). Collagen structure stability is closely associated with amino acid composition and sequence and thus, the most up-regulated amino acids potentially take part in enhanced bone formation in GDD (30). The metabolic pathways possibly influenced by *Ano5^Cys360Tyr^* mutation were explored using metabolite enrichment analysis. A total of 44 metabolic pathways were altered, including pyrimidine metabolism, steroid biosynthesis, parathyroid hormone synthesis, secretion and action, endocrine and other factor-regulated calcium reabsorption, and mineral absorption (**Figure 2C**). The five most enriched metabolic pathways were highlighted in **Table 2**, in which the activated metabolism of pyrimidine is positively associated with bone cell growth due to its capacity to provide energy and most metabolic substrates for living organisms (31). Additionally, obvious elevation of calcitriol, belonging to alcohol and amines class of metabolites, involved in endocrine and factor-regulated calcium reabsorption, which also plays a vital role in bone regeneration.

**Table 2:** The top five altered metabolic pathways in *Ano5^Cys360Tyr^* mCOBs compared to wild type mCOBs.

KEGG pathway	*p*-value	Differential metabolites
Thermogenesis	0.016	norepinephrine, 17β-estradiol
Chemical carcinogenesis- receptor activation	0.016	norepinephrine, 17β-estradiol
Endocrine and other factor-regulated calcium reabsorption	0.016	17β-estradiol, calcitriol
Pyrimidine metabolism	0.027	uridine, β-alanine, cytidine, cytosine,
Caffeine metabolism	0.045	caffeine, xanthine

### 3.2 Altered mCOB transcriptomes in *Ano5^Cys360Tyr^* mice

**Figure 3:**
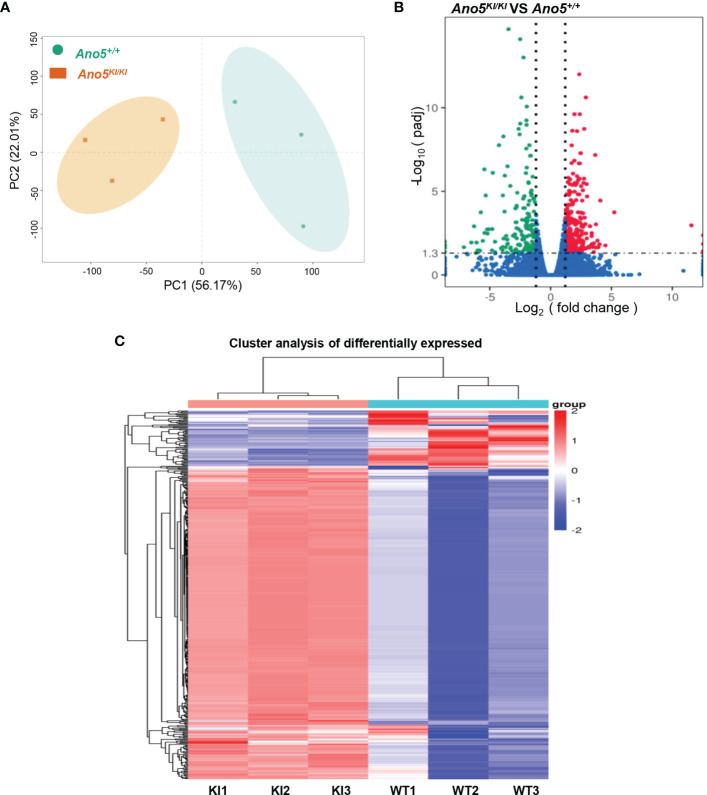
Differential gene expression analysis of *Ano5^KI/KI^* (KI) and *Ano5^+/+^* (WT) groups. **(A)** PCA of DEGs profiles. PC1 and PC2 indicate the degree of interpretation of the principal component analysis model, with the first and second ranked principal components, respectively; **(B)** Volcano plots of mRNA expression levels. The volcano map is drawn with −log_10_(*p*_adj_) as the vertical axis and log_2_(fold change) as the horizontal axis. The red dots mean up-regulated mRNAs and the blue dots represent down-regulated mRNAs, and the mRNAs with no significant changes were shown in grey dots; **(C)** Hierarchical heatmap of DEG profiles. Each row represents a sample, and each column represents an mRNA transcript. The color scale (from bright blue to bright red) indicates increasing transcript levels.

In order to explore the deep root of impaired *Ano5^KI/KI^* osteoblast metabolism, the gene expression profiles of mCOBs after 14 days of osteogenic induction from *Ano5^+/+^* and *Ano5^KI/KI^* mice were evaluated with RNA-seq. PCA revealed a clear distribution in gene expression profiles between *Ano5^+/+^* and *Ano5^KI/KI^* mCOBs (**Figure 3A**). A total of 407 differentially expressed genes (DEGs) were screened out based on the criteria of fold change >1.2 and *p-*adj < 0.05. Volcano plots indicated that 239 DEGs were up-regulated and 168 were down-regulated compared to the wild type group (**Figure 3B**). In addition, the relative levels of DEGs in *Ano5^+/+^* and *Ano5^KI/KI^* mCOBs were visualized with a heat map, which also revealed an obviously different clustering (**Figure 3C**). Based on log_2_Fc, the top 25 up-regulated DEGs were highlighted in **Table 3**. *Mki67* is a standard marker of cellular proliferation and exhibited an obvious elevation. *Cell Division Cycle 25C* (*Cdc25c*) participates in regulating the G2/M transition and mediating DNA damage repair through activating the cyclin B1 (CACNB1)/CDK1 complex (27, 32, 33). *Gad2* encodes glutamic acid decarboxylase that is responsible for catalyzing γ-aminobutric acid (GABA) production that has a positive effect on proliferation and osteogenic differentiation of mesenchymal stem cells (34).Simultaneously, **Table 4** illustrated the top 25 down-regulated DEGs. Notably, *Cytokine-like 1* (*Cytl1*) depletion exhibited a high bone mass phenotype due to enhanced osteogenesis and inhibited osteoclast activity (35). *Sost* is a potent negative regulator of bone formation by means of competitive interaction with the low‐density lipoprotein receptor‐related protein (LRP) 5/6 to antagonize WNT signaling (36, 37).

**Table 3:** The 25 most up-regulated genes in *Ano5^Cys360Tyr^* mCOBs.

Gene name	Description	Log_2_Fc	*P* adj
*Xist*	inactive X specific transcripts	11.573	0.00109
*Stmn4*	stathmin-like 4	5.2296	0.000181
*H2-M9*	histocompatibility 2, M region locus 9	4.5009	0.017333
*Mmp25*	matrix metallopeptidase 25	4.0879	3.55E-05
*Map3k7cl*	Map3k7 C-terminal like	3.8515	0.008906
*Gad2*	glutamic acid decarboxylase 2	3.7249	0.000943
*Tgtp2*	T cell specific GTPase 2	3.7047	0.022499
*8030451A03Rik*	RIKEN cDNA 8030451A03 gene	3.6639	6.73E-08
*Gabra4*	gamma-aminobutyric acid (GABA) A receptor, subunit alpha 4	3.5494	0.01283
*Ccl8*	chemokine (C-C motif) ligand 8	3.5479	0.042929
*Grm1*	glutamate receptor, metabotropic 1	3.4747	0.021504
*Thsd7b*	thrombospondin, type I, domain containing 7B	3.3835	0.014328
*Cdc25c*	cell division cycle 25C	3.3771	0.003147
*Nek2*	NIMA (never in mitosis gene a)-related expressed kinase 2	3.3679	0.004813
*Lrrc7*	leucine rich repeat containing 7	3.3017	0.017874
*Neurl1b*	neuralized E3 ubiquitin protein ligase 1B	3.2383	0.001239
*Kif18b*	kinesin family member 18B	3.1620	0.01978
*Cdh5*	cadherin 5	3.0010	0.023415
*Lrtm1*	leucine-rich repeats and transmembrane domains 1	2.9941	2.03E-05
*Kif2c*	kinesin family member 2C	2.9682	0.006596
*B3gnt5*	UDP-GlcNAc:betaGal beta-1,3-N-acetylglucosaminyltransferase 5	2.9611	3.71E-06
*Serpinb9b*	serine peptidase inhibitor, clade B, member 9b	2.8965	0.003295
*Heyl*	hairy/enhancer-of-split related with YRPW motif-like	2.8959	2.42E-11
*Mki67*	antigen identified by monoclonal antibody Ki 67	2.8618	0.027473
*Clca3a2*	chloride channel accessory 3A2	2.8545	0.000911

**Table 4:** The 25 most down-regulated genes in *Ano5^Cys360Tyr^* mCOBs.

Gene name	Description	Log_2_Fc	*P* adj
*Chad*	chondroadherin	-7.1308	0.021914
*Cytl1*	cytokine-like 1	-6.5204	0.011573
*Sptlc3*	serine palmitoyltransferase, subunit 3	-6.1409	0.049451
*Otos*	otospiralin	-6.0901	0.011543
*Coch*	cochlin	-5.9929	0.033559
*Gpha2*	glycoprotein hormone alpha 2	-5.5777	0.009241
*A230077H06Rik*	RIKEN cDNA A230077H06 gene	-5.4368	0.00306
*Scin*	scinderin	-5.3358	1.78E-05
*Colgalt2*	collagen beta(1-O) galactosyltransferase 2	-5.2200	0.001891
*Ucma*	upper zone of growth plate and cartilage matrix associated	-5.1882	0.003354
*Vstm2b*	V-set and transmembrane domain containing 2B	-5.0822	0.001859
*Hrk*	harakiri, BCL2 interacting protein	-4.8185	0.034058
*Foxo6os*	forkhead box O6, opposite strand	-4.7986	0.001807
*Gdf5*	growth differentiation factor 5	-4.5709	0.043416
*Adamts18*	a disintegrin-like and metallopeptidase with thrombospondin type 1 motif, 18	-4.0760	0.025281
*Col9a3*	collagen, type IX, alpha 3	-3.9519	0.03455
*Col11a2*	collagen, type XI, alpha 2	-3.9432	0.021056
*Stk32b*	serine/threonine kinase 32B	-3.9280	1.77E-05
*Wfdc2*	WAP four-disulfide core domain 2	-3.8843	5.23E-09
*Sost*	sclerostin	-3.8716	0.006775
*Dlx6*	distal-less homeobox 6	-3.7888	0.000164
*Ppp1r1b*	protein phosphatase 1, regulatory inhibitor subunit 1B	-3.7649	1.15E-06
*Rab11fip4*	RAB11 family interacting protein 4 (class II)	-3.5953	0.046581
*Dlx6os1*	distal-less homeobox 6, opposite strand 1	-3.5645	0.00769
*Slc4a11*	solute carrier family 4, sodium bicarbonate transporter-like, member 11	-3.5462	0.014282

### 3.3 GO and KEGG enrichment analysis

**Figure 4:**
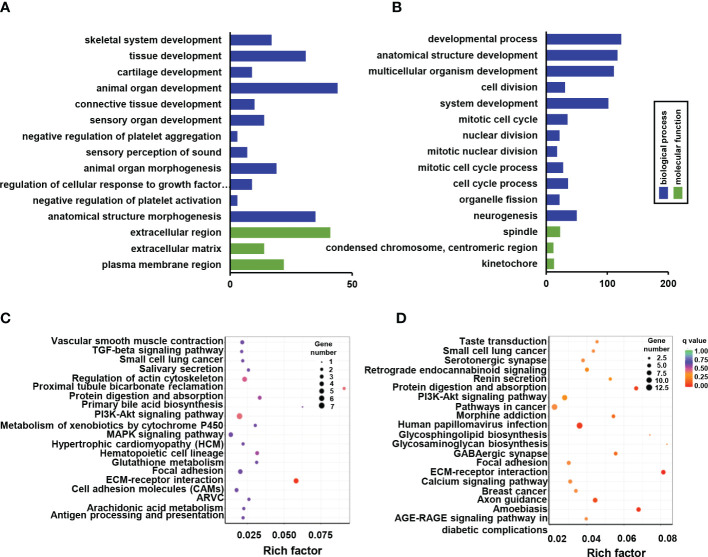
GO and KEGG pathway enrichment of DEGs between *Ano5^+/+^* and *Ano5^KI/KI^* groups. **(A, B)** GO enrichment analysis of down-regulated DEGs **(A)** and up-regulated DEGs **(B)**, including the categories of biological process (BP) and molecular function (MF); **(C, D)** KEGG enrichment analysis of down-regulated DEGs **(C)** and up-regulated DEGs **(D)**.

GO enrichment analysis was used to enrich the function of differentially expressed genes. Only four down-regulated categories were statistically enriched, including skeletal system development, tissue development, extracellular region, and extracellular matrix development (**Figure 4A**). While 251 significantly up-regulated terms were identified and mainly involved in system development, cell cycle process, and nuclear division (**Figure 4B**). Functional pathway analysis was also conducted based on KEGG enrichment, although only part of the ECM-receptor interaction signaling pathways exhibited significant down-regulation (**Figure 4C**). It is worth noting that prominent signaling pathways with elevated tendency were closely associated with osteogenic alteration of *Ano5^KI/KI^* mCOBs, including ECM-receptor interaction, protein digestion and absorption, and calcium signaling pathway (**Figure 4D**).

### 3.4 Integrated analysis of transcriptome and metabolome profiles

**Figure 5:**
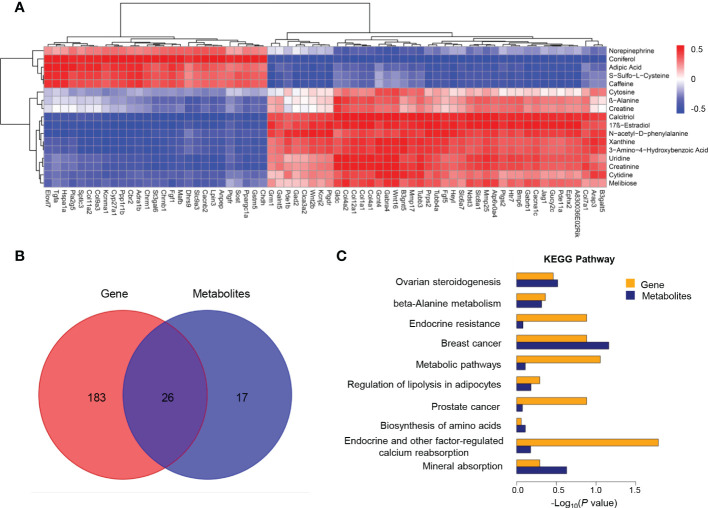
Co-analysis of mCOB transcriptomes and metabolomes. **(A)** Heat map showing the Spearman correlation hierarchical cluster analysis of DEGs and differential metabolites. Horizontal direction representing genes and the vertical axis showing metabolites; **(B)** Venn diagram illustrating the overlap of pathways associated with DEGs and metabolites; **(C)** The top 10 shared KEGG pathways. The horizontal length reflecting FDR value. Blue color for genes and orange for metabolites.

In order to better understand the correction patterns of among significantly differentiated genes and metabolites, cluster analysis was performed (**Figure 5A**). Functional enrichment analysis revealed 26 shared pathways of DEGs and differential metabolites as illustrated by the Veen diagram (**Figure 5B**). The ten most enriched KEGG pathways were particularly scrutinized to reveal critical metabolic processes differing between *Ano5^KI/KI^* and *Ano5^+/+^* mCOBs (**Figure 5C**). The analysis indicated that *Slc8a1*, accompanied by 17β-estradiol and calcitriol that participate in endocrine and other factor-regulated calcium reabsorption. Furthermore, β-alanine metabolism, biosynthesis of amino acids, and mineral absorption may be key processes associated with enhanced osteogensis in GDD.

### 3.5 Effect of *Ano5^Cys360Tyr^* mutation on mCOB proliferation

**Figure 6:**
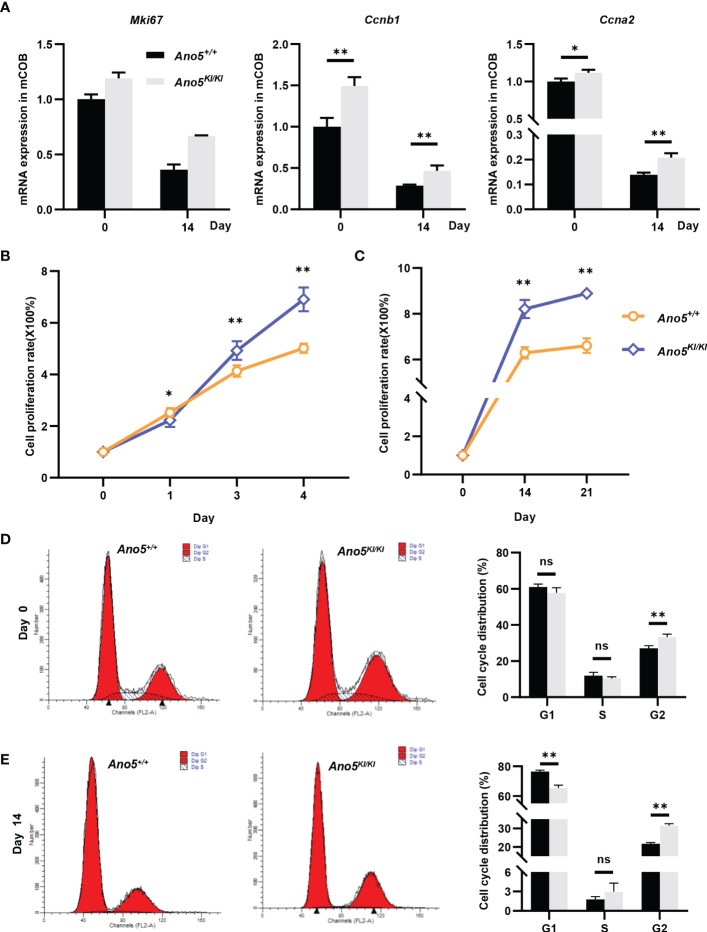
Cellular prolieration and cell cycle analysis in *Ano5^+/+^* and *Ano5^KI/KI^* mCOBs. **(A)** Relative expression of *Mki67*, *Ccnb1*, and *Ccna2* in *Ano5^KI/KI^* compared with *Ano5^+/+^* mCOBs at days 0 and 14 after osteogenic induction; **(B, C)** Cellular proliferation was evaluated using CCK-8 assays at days 1, 3, and 4 without os Please expand the term "ns" in Figures 6, 7 if applicable Please expand the term "ns" in Figures 6, 7 if applicableteoblast differentiation **(B)** and at days 14 and 21 with osteogenic induction **(C)**; **(D, E)** Cell cycle distributions were assessed with flow cytometry at days 0 **(D)** and 14 after osteogenic differentiation **(E)**. Data were analyzed using Student’s *t-*tests or one-way ANOVA tests with Dunnett’s multiple comparison tests. ns, no significance; *p < 0.05; **p < 0.01.

GO analysis implied that cell cycle and nuclear division were abnormally activated in the *Ano5^KI/KI^* mCOBs. Therefore, subsequent studies focused on the effects of the *Ano5^Cys360Tyr^* mutation on cell proliferation. In addition to *Mki67*, *cyclin A2* (*Ccna2*), an essential regulator of the G1/S and G2/M transition mediating binding and activating CDK2 (38) was up-regulated in *Ano5^KI/KI^* mCOBs based on RNA-seq analysis. Likewise, *Ccnb1* that forms a complex with CDK1 to promote the transition from the G2 phase of cell cycle to mitosis was also increased. qRT-PCR further confirmed that the p.Cys360Tyr mutation in *Ano5* enhanced expression of those genes in both preosteoblasts at day 0 and mature mCOBs at day 14 after osteogenic induction (**Figure 6A**). Interestingly, the upward trend was more obvious at day 14. Subsequently, we are driven to disclose the proliferation ability of GDD-related osteoblasts and CCK8 indicated that either preosteoblasts or mature mCOBs from *Ano5^KI/KI^* mice grew faster than those from wildtype mice (**Figures 6B, C**). To explore whether GDD-induced hyperproliferation was associated with cell cycle alternation, we detected the cell cycle distribution of mCOB from *Ano5^+/+^* and *Ano5^KI/KI^* mice using flow cytometry to analyze cellular DNA content. mCOBs from *Ano5^KI/KI^* mice displayed greater numbers of cells in the G2 phase compared with the wildtype group at day 0 (**Figure 6D**). Furthermore, *Ano5^KI/KI^* mature osteoblasts exhibited decreased cell proportions in the G1 phase and significantly increased abundances of cells in the G2 phase (31.56 ± 0.94 vs. 21.74 ± 0.61) (**Figure 6E**). In conclusion, these observations suggested that increased osteogenesis in GDD can be partially explained by enhanced *Ano5^KI/KI^* osteoblast proliferation.

### 3.6 Calcium-related alternation in *Ano5^Cys360Tyr^* osteoblasts

**Figure 7:**
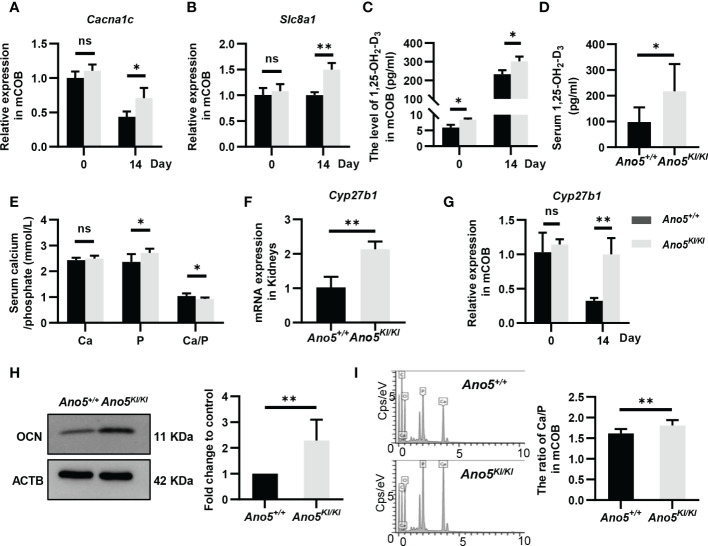
Detection of calcium-related genes and calcium content in mineral nodules. **(A, B)** Relative expression of *Cacna1c* and *Slc8a1* in *Ano5^KI/KI^* mCOBs at days 0 and 14 normalized to levels in *Ano5^+/+^* cells; **(C)** Calcitriol levels (pg/ml) were measured by ELISA in intracellular of mCOBs from *Ano5^+/+^* and *Ano5^KI/KI^* mice at days 0 and 14 after osteogenic induction; **(D, E)** Serum calcitriol, calcium, phosphate contents in *Ano5^+/+^* and *Ano5^KI/KI^* male mice (n=10) at 16 weeks of age; **(F)** qRT-PCR analysis of *Cyp27b1* in kidneys of *Ano5^+/+^* and *Ano5^KI/KI^* male mice (n=4); **(G)** Relative expression of *Cyp27b1* in *Ano5^KI/KI^* mCOBs at days 0 and 14 normalized to levels in *Ano5^+/+^* cells; **(H)** Immunoblotting and quantitative analysis of OCN in *Ano5^KI/KI^* compared with *Ano5^+/+^* mCOBs after 14 days of osteogenic induction; **(I)** The distribution of elements detected by energy dispersive X-ray spectroscopy and ratio of calcium and phosphate in the mineralized matrix of *Ano5^+/+^* and *Ano5^KI/KI^* cultures. Data were analyzed using *t-*tests or one-way ANOVA tests with Dunnett’s multiple comparison tests. ns, no significance; *p < 0.05; **p < 0.01.

GDD lesions are mainly characterized by disturbances in bone regeneration, wherein calcium is a structural inorganic component and plays a prominent role in bone matrix mineralization and osteoblast differentiation. Accordingly, subsequent studies are needed to evaluate calcium signaling in this context. Both transcriptomics and metabolomics analysis suggested that *Ano5^Cys360Tyr^* mutation could influence calcium reabsorption and calcium signaling pathways. Further, RNA-seq data also indicated significant up-regulation of *solute carrier family 8 member A1* (*Slc8a1*), *calcium voltage-gated channel subunit alpha 1 C* (*Cacna1c*), *Htrt1*, *Grm1*, *Pde1b*, *Erbb3*, *Pdgfra*, and *Ptk2b* combined with down-regulation of *Chrm1*, *Adra1b*, and *Ptgfr*; all resulting in disturbed calcium homeostasis (**Supplementary Table 2**). *Cacna1c* is of particular interest, because a specific gain-of-function mutation (G406R) in its encoded protein is responsible for Timothy Syndrome (TS) that is clinically manifested by small teeth and dysmorphic facial features beyond classical cardiac arrhythmia (39). qRT-PCR indicated that the *Ano5^Cys360Tyr^* mutation promoted the expression of *Cacna1c* at days 14 after osteoblast differentiation, although no significant difference was observed at day 0 (**Figure 7A**). In addition, *Slc8a1* was upregulated in *Ano5^Cys360Tyr^* mature mCOBs, which participates in calcium transport to the extracellular depending on sodium (Na^+^) concentration gradients. These trends were further verified by qRT-PCR analysis (**Figure 7B**). It has been reported that *Slc8a1* is regulated by calcitriol also known as active 1,25(OH)_2_D (1,25-dihydroxy vitamin D), was classically involved in bone homeostasis. An ELISA experiment was further performed and revealed obvious intracellular calcitriol elevation in *Ano5^KI/KI^* mCOBs at days 0 and 14, even though the supernatants did not exhibit significant differences (**Figure 7C** and **Figure S3**). Calcitriol also plays an important role in the systemic circulation of calcium and phosphate (40). *Ano5^KI/KI^* mice manifested a massive up-regulation of serum calcitriol (217.523 ± 98.963 pmol/ml) compared with *Ano5^+/+^* mice (98.026 ± 53.298 pmol/ml) and this was combined with slight phosphate increases, which indicated high bone turnover (**Figures 7D, E**). Calcitriol is synthesized by the mitochondrial enzyme 25-hydroxyvitamin D-1a-hydroxylase encoded by *CYP27B1* using hydroxylation of 25(OH)D_3_ as the substrate (41, 42). Meaningfully, qRT-PCR showed that *Cyp27b1* was significantly elevated in the kidneys of *Ano5^KI/KI^* mice and a three-fold increase in *Cyp27b1* mRNA accumulation was also observed in *Ano5^Cys360Tyr^* mCOBs after 14 days of osteoblast differentiation, but not at day 0 (**Figures 7F, G**). Some studies have reported that calcitriol and Cyp27b1 cooperatively regulate OCN during osteogenic differentiation (43, 44). Consistently, the protein levels of OCN, the most abundant non-collagenous bone matrix protein that is specifically synthesized by osteoblasts, was elevated in differentiated *Ano5^KI/KI^* mCOBs (**Figure 7H**).

The fact of calcium signaling alteration provides a reasonable explanation for lower levels of free calcium in the cytoplasm of *Ano5^Cys360Tyr^* mCOBs compared to *Ano5^+/+^* mCOBs as we previously observed (29). In the present study, SEM-EDS were further performed to measure the contents of calcium and phosphorous in mineralized nodules. The ratio of calcium versus phosphorous was higher in *Ano5^Cys360Tyr^* than *Ano5^+/+^* mCOBs (**Figure 7I**). This phenomenon implies that *Ano5^Cys360Tyr^* mutation may perturb calcium balance between extracellular environments and the cytoplasm, leading to enhanced bone formation that can be attributed to up-regulation of calcitriol and *Cyp27b1* expression.

## 4 Discussion

We previously successfully built a knock-in mouse model equivalent of the ANO5 p.Cys360Tyr mutation, which displayed some bone phenotypes consistent with GDD patients, including gross thickening of cortical diaphysis, increasing bone fragility, and enhanced serum ALP levels. Most importantly, mCOB cultures from *Ano5^Cys360Tyr^* mice exhibited aberrant enhancement of osteogenic differentiation and matrix mineralization (29). Various metabolic pathways are highly coordinated that participate in bone formation and maintain bone homeostasis. However, it remains unclear how the ANO5 dominant mutation excessively activates bone formation. Systematic observations on differential gene expression and metabolic pathways were conducted between wild type and *Ano5^KI/KI^* mature mCOBs after 14 days of osteogenic induction to establish a comprehensive understanding of the molecular mechanisms underlying enhanced osteogenesis in GDD.

GDD-related ANO5 mutations result in disturbed bone lesions and thus, calcium metabolism was a focus of our present study due to its role in the fate of osteoblasts and the formation of bone matrix. Bone is the largest store for calcium and exists in the form of calcium phosphate hydroxyapatite [Ca_10_(PO_4_)_6_(OH)_2_]. Consequently, calcium has long been regarded as a fundamental component of preventing and treating osteoporosis (45). A previous study revealed that the C356R and C356G mutations of ANO5 induced rounded cell morphologies that could occur due to excessive calcium release from the endoplasmic reticulum (4). Additionally, an *Ano5* deficiency disturbs the ability of endoplasmic reticulum to clear injury-triggered calcium accumulation, which is associated with blocked plasma membrane repair and serves as an important pathological mechanism dedicated to muscular dystrophies - limb girdle muscular dystrophy type 2L (LGMD2L) and Miyoshi myopathy type 3 (MMD3) caused by ANO5 recessive mutations (46, 47). However, the direct effects of *ANO5* mutation on calcium homeostasis in osteoblasts have not been clarified. The SEM-EDS analyses of this study indicated a higher ratio of calcium versus phosphate in mineral nodules from *Ano5^KI/KI^* mCOB cultures that were accompanied by decreased free calcium levels in the cytoplasm. Although there is upregulation of some genes responsible for calcium influx, including *Cacna1c* and *Pdgfra*, we speculated these dynamics could be mainly attributed to upregulation of *Slc8a1*, one of the vital regulators of Ca^2+^ homeostasis, which could facilitate two calcium efflux and three Na^+^ influx depending on gradients of transported ions and membrane potentials. It is reported that ANO6, the closest paralog of ANO5, activates SLC8A1 to promote osteogenesis, in which the potential imbalance caused by SLC8A1 requires ANO6-mediated Ca^2+^ activated anion currents to compensate (48), and thus we speculated that an ionic equilibrium in GDD-related osteoblasts may be achieved by coordination of enhanced *Slc8a1* and ANO5 gain of function of Ca^2+^ dependent outwardly rectifying ionic currents as reported by Anna using HEK293T cell lines (7). However, the regulatory outcome of *Ano5^Cys360Tyr^* mutation in calcium homeostasis requires more comprehensive exploration.

Notably, our integrated analysis indicated that calcitriol, estradiol, and *Slc8a1*, cooperatively interact to disturb calcium homeostasis in *Ano5^Cys360Tyr^* mCOBs. Both metabolomics and ELISA analysis demonstrated that the p.Cys360Tyr mutation led to massive elevation of calcitriol levels in mCOBs. While we failed to collect biochemical index of 25(OH)D_3_ from in the Han GDD patient, *Ano5^KI/KI^* mice manifested higher calcitriol levels in serum and *Cyp27b1* expression in kidneys than wild type mice, which indicated that the generation of calcitriol from 25(OH)D_3_ was facilitated. Importantly, serum 25(OH)D_3_ concentration, one of the most reliable biomarkers of calcitriol metabolic status, is decreased in GDD patients caused by p.Ser500Phe and p.Arg597Ile mutations in *ANO5*, which is a manifestation of increased calcitriol synthesis (14, 49). It is worth noting that calcitriol is involved in calcium reabsorption and mineral deposition by activating calcium channels so as to play a vital role in bone regeneration. Calcitriol can stimulate the absorption of calcium and phosphate in the small intestine, leading to generation of optimal circumstances for matrix mineralization (43, 50–52). More and more scholars have recommended adequate 1,25(OH)_2_D_3_ uptake to prevent osteoporosis, wherein its anti-aging mechanism depends on promoting matrix mineralization and preventing bone resorption (53). In addition to bone formation, calcitriol also plays a critical role in osteoclast differentiation and even bone resorption, which relies on whether it regulates osteoclastogenesis in a direct manner or mediated by osteoblast-secreted RANKL and osteoprotegerin (OPG) (54). Based on the higher ratio of OPG/RANKL and impaired osteoclastogenesis in *Ano5^KI/KI^* mice compared to wild type individuals, it is reasonable to speculate that increased calcitriol is crucial for bone turnover underlying GDD. In order to explore the mechanisms contributing to excessive production of calcitriol caused by the *Ano5^Cys360Tyr^* mutation, we detected the expression levels of *fibroblast growth factor 23* (*Fgf23*) that is primarily secreted by osteoblasts and osteocytes and regulates phosphate homeostasis and calcitriol metabolism (43), while result showed both its expression in mCOB and serum FGF23 level were comparable between *Ano5^+/+^* and *Ano5^KI/KI^* group (**Figure S4**). In addition to FGF23, elevated serum phosphate may be attributed to abnormal metabolism of calcitriol and parathyroid hormone, and the specific mechanism remains to be further explored. Moreover, elevated *Cyp27b1* expression was observed in mature *Ano5^KI/KI^* osteoblasts, which is identical to previous reports that the overexpression of CYP27B1 in osteoblasts leads to increased bone volume, consistent with the bone phenotype of *Ano5^KI/KI^* mice. Both calcitriol and Cyp27b1 could stimulate the expression of *25-hydroxyvitamin D 24-hydroxylase* (*Cyp24a1*) (44), which was consistently up-regulated in both *Ano5^KI/KI^* osteoblasts at day 14 and kidneys of *Ano5^KI/KI^* mice to attenuate the excessive calcitriol activity (**Figure S5**). Interestingly, *Ano5^KI/KI^* mature osteoblasts exhibited profound increases in OCN protein levels that is a downstream factor of calcitriol and located in intrafibrillar or interfibrillar regions of mineralized nodules to participate in the formation of crystal thickness, shape, and orientation. More importantly, OCN is a osteoblast-specific secreted hormone, which stimulates the production and secretion of insulin, but also favors glucose and fatty acid homeostasis to provide an an interaction point between calcium and energy metabolism (55).

Apart from osteogenic differentiation, calcitriol has a crucial impact on cellular growth. Many lines of evidence have indicated that 1,25(OH)_2_D_3_ could contribute to promoting proliferation of bone ancestor cells, such as stem cells from the apical papilla and human bone marrow mesenchymal stem cells (43, 56). Additionally, ANO5 is known to participate in proliferation of myoblasts and some cancer cells (57). Interestingly, *Ano5^KI/KI^* mCOBs exhibited an obvious enhancement in proliferation ability regardless of whether osteogenesis was induced, as was consistently observed in *Ano5* knockout osteoblasts (data not shown). All somatic cells proliferate determined by the cell cycle *via* mitotic processes that were correspondingly up-regulated in *Ano5^KI/KI^* mCOBs, as suggested by GO analysis. Nevertheless, cellular division is a complex process that is tightly controlled by the coordinated action of multiple molecular mechanisms. In addition to *Mki67*, RNA-seq revealed that regulators of the cell cycle like *Ccna2*, *Ccnb1*, *Bub1b*, and *Cdc25c* were significantly elevated in *Ano5^KI/KI^* mCOBs compared to *Ano5^+/+^* mCOBs. Accordingly, *Ano5^KI/KI^* mCOBs at days 0 and 14 displayed significantly prolonged G2 phases accompanied by elevated *Ccna2* and *Ccnb1* expression that promotes the transition from the G2 phase to mitosis (32, 38). A slightly declined G1 phase in *Ano5^KI/KI^* mature mCOBs may be mediated by *Ccna2* up-regulation that is also responsible for the G1/S transition. The heightened activity of cellular growth was supported by plentiful metabolic substrates, as indicated by heightened purine and pyrimidine metabolism that could provide necessary components for RNA and DNA biosynthesis (31). Over 10 purine antimetabolites have been approved for cancer treatments, which provides potential prospects for GDD treatment (58).

Abundant energy-generating capacity is indispensable for osteoblasts to facilitate proliferation and differentiation. In addition to glucose utilization, long-chain fatty acid β-oxidation is also an important energy source for osteoblasts to promote bone formation. The clearly decreased expression levels of *Sost* as shown in RNA-seq results, indicated that β-oxidation of fatty acids may be excessively stimulated by the *Ano5^Cys360Tyr^* mutation that is mediated by activated WNT-LRP5 signaling (59). Notably, carnitine C4:DC is involved in the transport of acetyl-CoA into mitochondria to generate ATP and was highly enriched by approximately 10-fold in *Ano5^KI/KI^* mCOBs compared to *Ano5^+/+^* cultures. We also noticed that the p.Cys360Tyr mutation in *Ano5* exhibited a momentous impact on amino acid metabolism. β-alanine levels are positively correlated with high BMD in human (60) and were emerging in mCOBs from mice expressing the *Ano5^Cys360Tyr^* mutation. β-alanine exerts a key influence on bone formation primarily by promoting insulin production that further improves osteoblast proliferation, differentiation, and collagen synthesis. In addition to alanine, L-histidine also is a key component of the collagen spiral structure and metabolized by histidinol dehydrogenase using the substrate L-histidinol, which showed an increased tendency in *Ano5* knock-in mCOBs (30). Overall, the metabolic disorders of energy metabolism observed here provide avenues for exploring the mechanisms underlying GDD particularly including enhanced cell proliferation, aberrant matrix mineralization, and abnormal collagen arrangement in diaphysis.

## 5 Conclusion

To our knowledge, this is the first study to conduct a comprehensive multi-’omics integrative analysis to systematically assess the mechanisms underlying abnormal osteogenesis caused by GDD-related mutations using the *Ano5^Cys360Tyr^* mouse model. The data indicates that alterations of amino acid, fatty acid, and purine metabolism, combined with abnormal calcium-related signaling pathway may be responsible for promoting osteoblast proliferation and differentiation due to GDD-related mutations. Further investigations targeting key downstream molecular and signaling pathways of ANO5 would be promising avenues to identify effective therapeutic targets.

## Data availability statement

The datasets presented in this study can be found in online repositories. The names of the repository/repositories and accession number(s) can be found below: https://www.ncbi.nlm.nih.gov/, PRJNA899301.

## Ethics statement

The animal study was reviewed and approved by Institutional Animal Care and Use Committee of the Beijing Stomatological Hospital (the approval number: KQYY-201611-001).

## Author contributions

YH and HL were responsible for the conception and design of the study. HL, SL, and CM contributed data collection. YH, HL, and YL performed formal analysis. YH and HL drafted and revised the manuscript. All authors contributed to the article and approved the submitted version.
