# Supplementary material for: Integration of metabolomics and transcriptomics provides insights into enhanced osteogenesis in Ano5Cys360Tyr knock-in mouse model
Source: Front Endocrinol (Lausanne). 2023 Jan 20;14:1117111. doi: 10.3389/fendo.2023.1117111 (PMC9895949; doi:10.3389/fendo.2023.1117111)
Supplement: Supplementary file 1 [file DataSheet_1.docx]

Supplementary Material

## Supplementary Figures


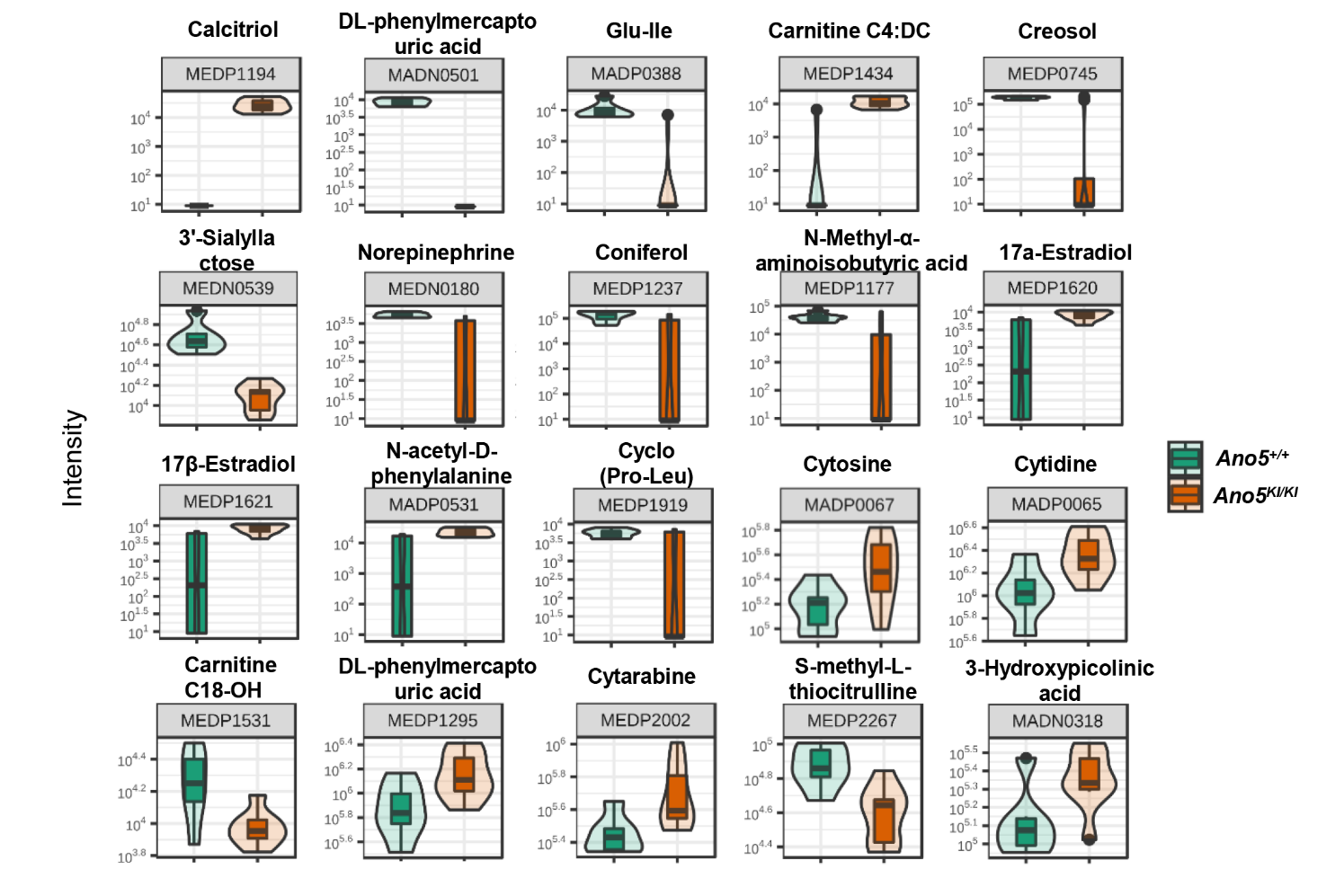


**Figure S1.** Violin diagram of the 20 most differential metabolites in *Ano5^KI/KI^* mCOBs (orange) compared with *Ano5^+/+^* mCOBs (green). The metabolite corresponding to the index is listed in Supplementary Table S1. The Y-axis shows raw intensity values. The box in the middle represents the quartile range and the thin extended black line represents the 95% confidence interval, while the black horizontal line in the middle of the box shows the median. The overall box indicates the distribution density of the raw data.


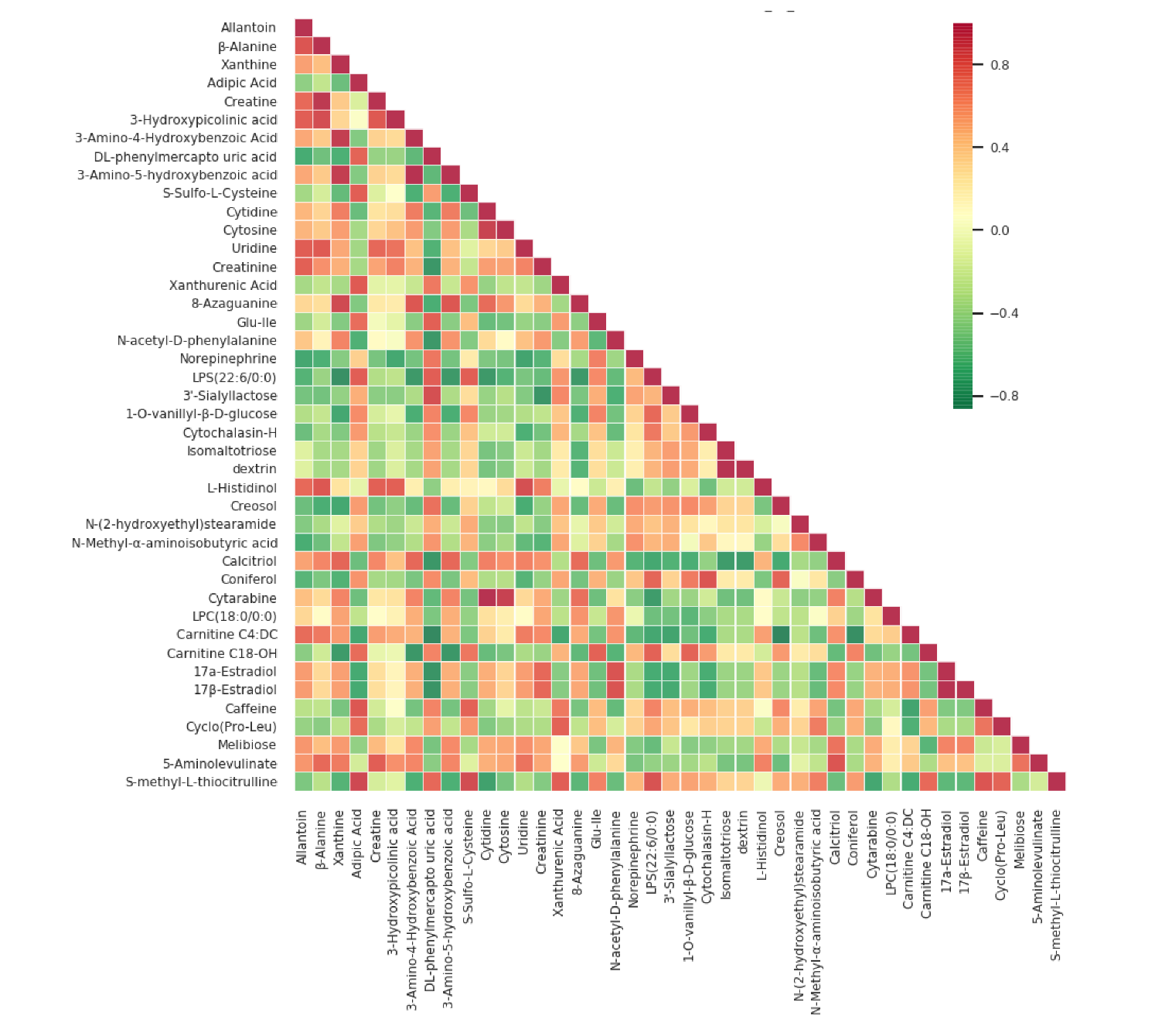


**Figure S2.** Pearson correlation analysis of differential metabolites. Pearson correlation analysis was used to analyze the metabolic associations among significantly different metabolites. Red color indicating a strong positive correlation and green indicating a clear negative correlation.


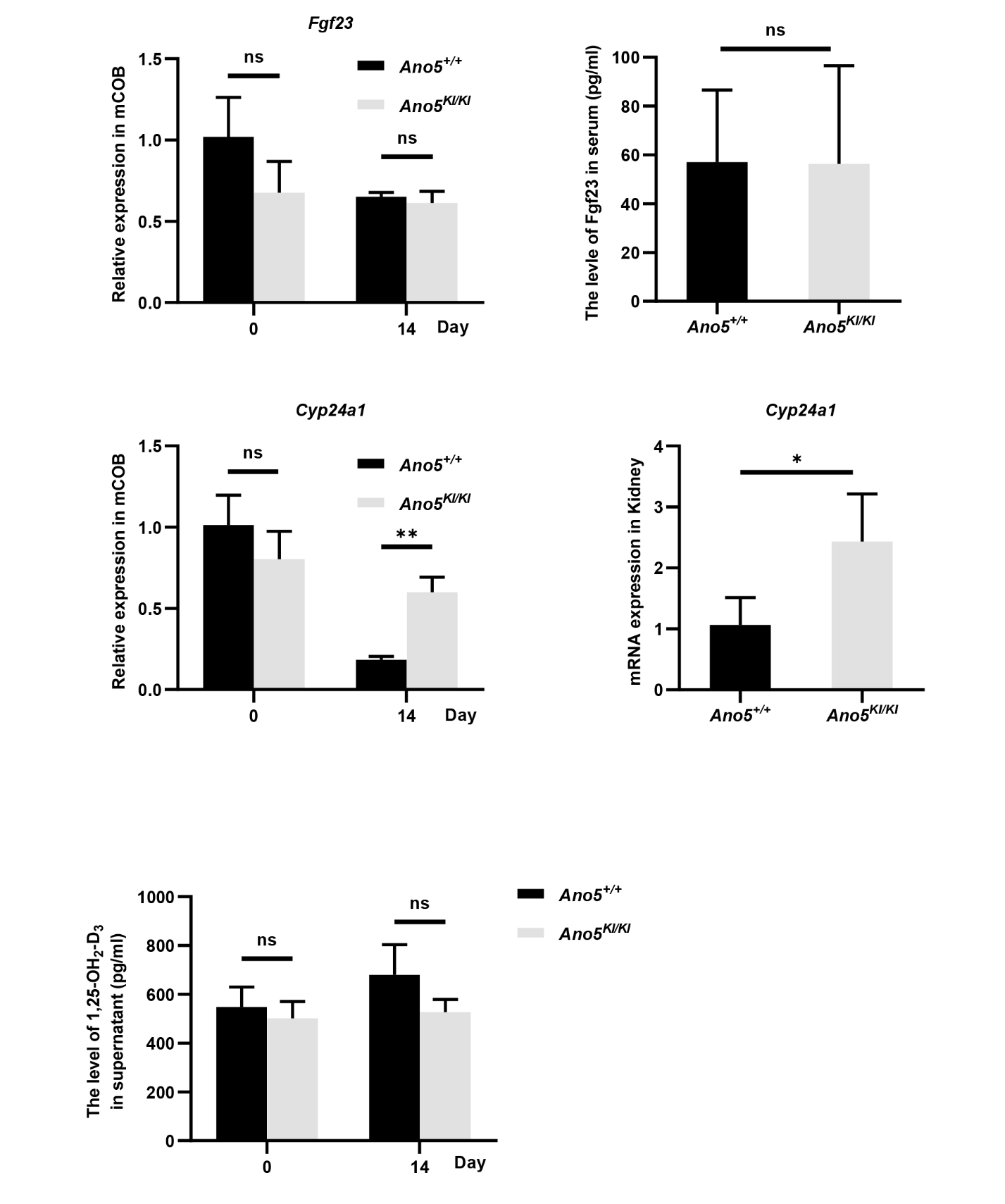


**Figure S3.** ELISA analysis of calcitriol in supernatant of *Ano5^+/+^* and *Ano5^KI/KI^* cultures at days 0 and 14. Data were analyzed using Student’s *t*-tests or one-way ANOVA tests with Dunnett's multiple comparison tests.


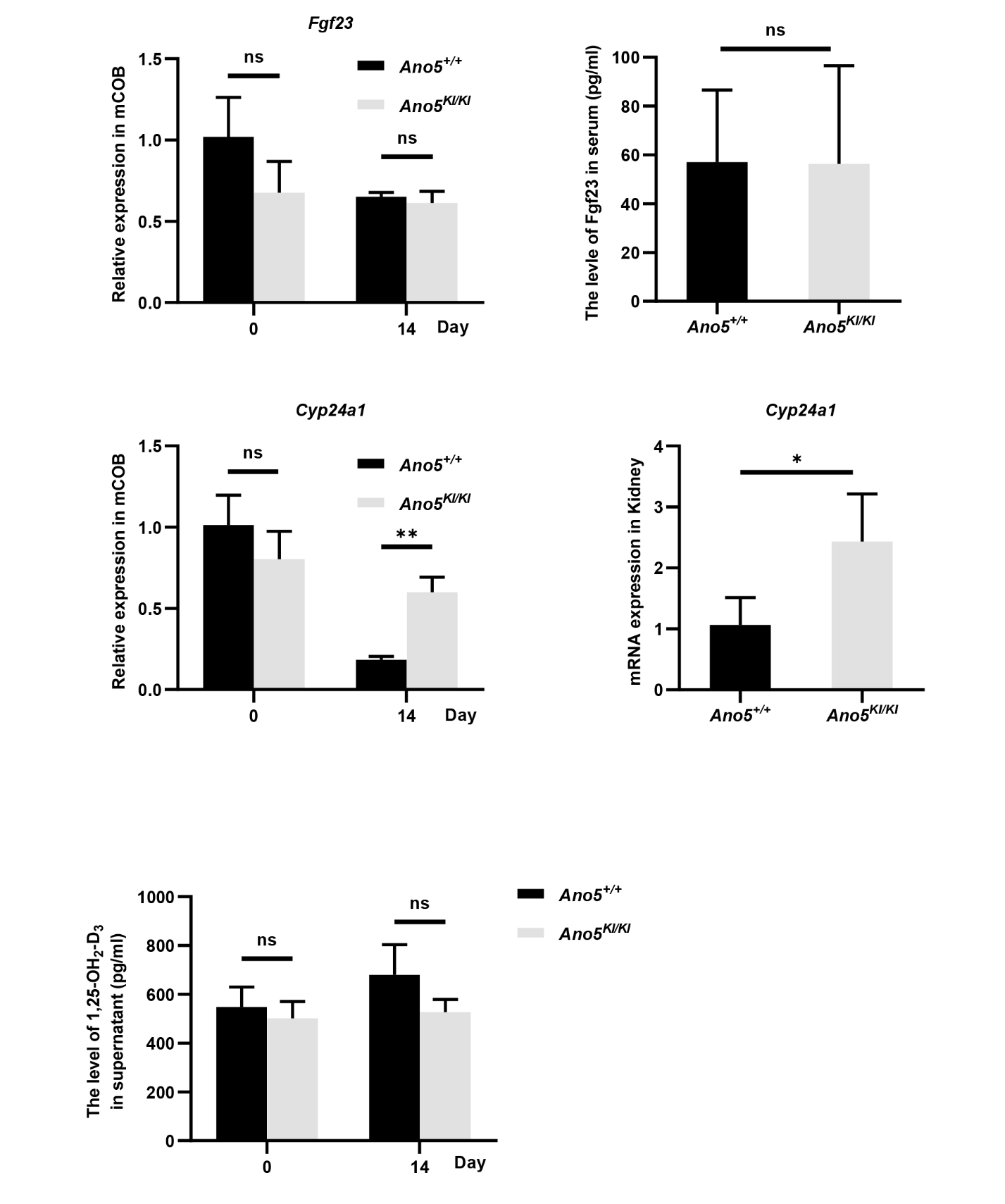


**Figure S4.** qRT-PCR detection of *Fgf23* at days 0 and 14 (left) and ELISA analysis of FGF23 levels in serum of 16-week-old *Ano5^+/+^* and *Ano5^KI/KI^* male mice (right). Data were analyzed using Student’s *t*-tests or one-way ANOVA tests with Dunnett's multiple comparison tests.


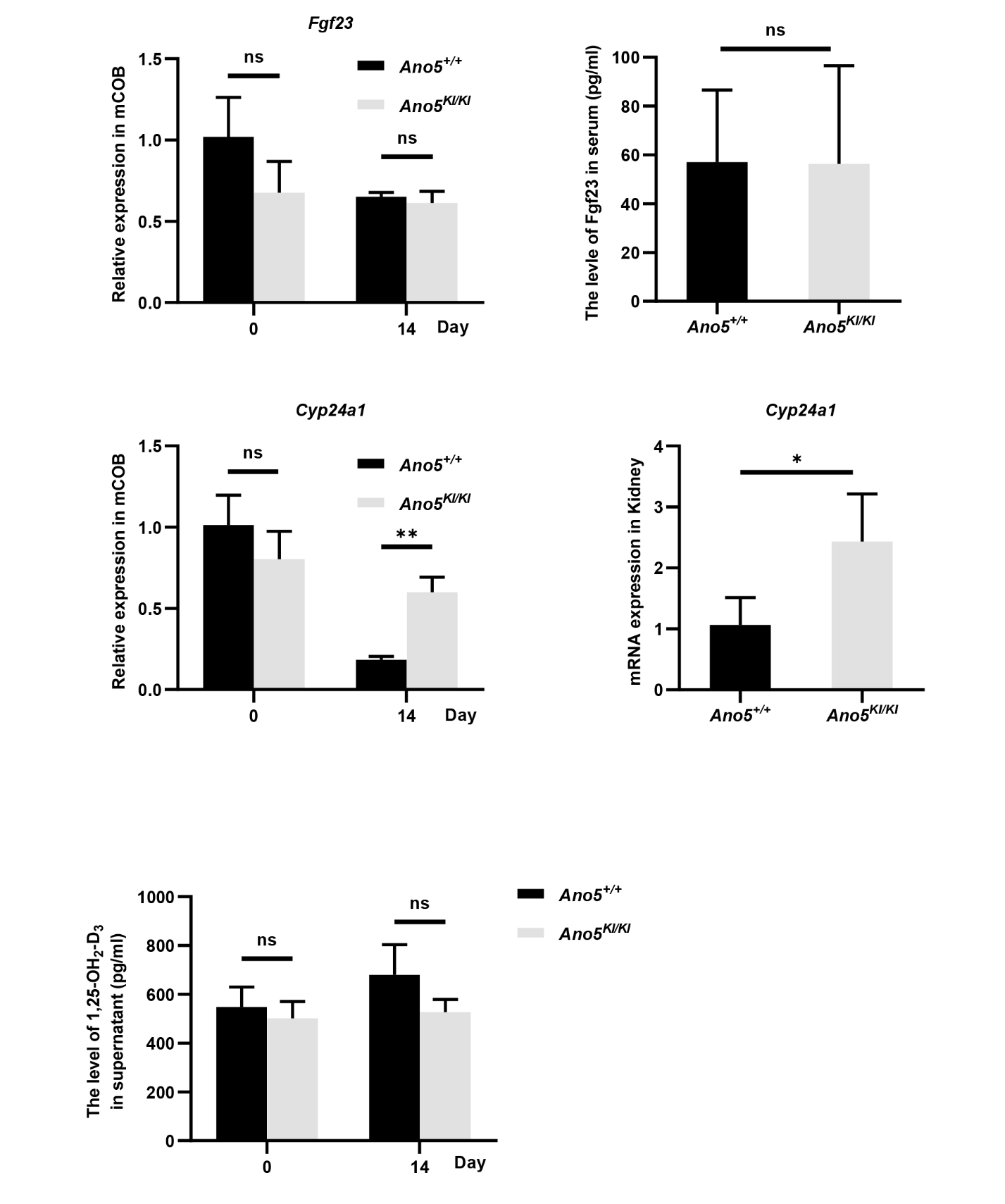


**Figure S5.** qRT-PCR detection of *Cyp24a1* in mCOB at days 0 and 14 (left) and in kidneys of 16-week-old *Ano5^+/+^* and *Ano5^KI/KI^* mice (right). Data were analyzed using Student’s *t*-tests or one-way ANOVA tests with Dunnett's multiple comparison tests. *: *p* < 0.05; **: *p* < 0.01.

1. **Supplementary Tables**

**Supplementary Table 1:** Primer sequences used for qRT-PCR assays.

| **Gene** | **Forward Primer** | **Reversed Primer** |
| --- | --- | --- |
| *Actb* | GTGACGTTGACATCCGTAAAGA | GCCGGACTCATCGTACTCC |
| *Slc8a1* | CTGGGGAAGATGACGATGAT | TTCTGTAGGTGGGACGAAGG |
| *Cyp27b1* | GCTGTGATGAAAGAAGTGTTGA | GTGGCATAGTGAGATAGGGAG |
| *Cyp24a1* | CTGCCCATTGCGTTCTGT | TCTTGATTTGGGGGTGAAAA |
| *Fgf23* | CAGCCAGGACCAGCTATCACCTA | AATGTTGCCCGTGGAGATCCATAC |
| *Cacna1c* | TGCTGTGTCTGACCCTGAAG | CGTCTTCCGGAAAGGGAATA |
| *Mki67* | GCCTCCTAATACACCACTGAA | GCCGTTCCTTGATGATTGTC |
| *Ccna2* | GCCTTCACCATTCATGTGGAT | TTGCTGCGGGTAAAGAGACAG |
| *Ccnb1* | AGTGCCTCTGAAAAGGGAAG | CTTCCTCCAGTTGTCGGAGA |

**Supplementary Table 2:** Genes with statistically differential expression levels involved in calcium signaling pathways in *Ano5^KI/KI^* compared with *Ano5^+/+^* mCOBs.

| **Gene name** | **Description** | **Type** | **log_2_Fc** | ***P* adj** |
| --- | --- | --- | --- | --- |
| *Slc8a1* | solute carrier family 8 | up | 2.1664 | 0.00000124 |
| *Cacna1c* | calcium channel, voltage-dependent, L type, alpha 1C subunit | up | 1.7695 | 0.00000275 |
| *Grm1* | glutamate receptor, metabotropic 1 | up | 3.4747 | 0.021504 |
| *Htr7* | 5-hydroxytryptamine (serotonin) receptor 7 | up | 2.2402 | 0.00227 |
| *Chrm1* | cholinergic receptor, muscarinic 1 | down | -2.7441 | 0.0000122 |
| *Adra1b* | adrenergic receptor, alpha 1b | down | -1.5031 | 0.00052508 |
| *Ptgfr* | prostaglandin F receptor | down | -2.1137 | 0.00052508 |
| *Erbb3* | erb-b2 receptor tyrosine kinase 3 | up | 1.2268 | 0.001105 |
| *Pde1b* | Phosphodiesterase 1B, Ca^2+^-calmodulin dependent | up | 1.7821 | 0.031665 |
